# The Effect of Afforestation on Soil Moisture Content in Northeastern China

**DOI:** 10.1371/journal.pone.0160776

**Published:** 2016-08-11

**Authors:** Yitong Yao, Xuhui Wang, Zhenzhong Zeng, Yongwen Liu, Shushi Peng, Zaichun Zhu, Shilong Piao

**Affiliations:** Sino-French Institute for Earth System Science, College of Urban and Environmental Sciences, Peking University, Beijing 100871, China; Chinese Academy of Forestry, CHINA

## Abstract

Widespread afforestation programs sequester carbon from the atmosphere and mitigate the rising of atmospheric carbon dioxide (CO_2_). Meanwhile, afforestation carbon sequestration may cost soil water. However, changes in soil moisture content (SMC) after large-scale afforestation or reforestation have rarely been quantified. In this study, we measured changes in SMC following afforestation using a paired plots method with data from 757 plots in Northeastern China. We found a marginally significant decline in soil moisture content of the top 1-m soil (SMC_0-1m_) after afforestation (P = 0.08) at the regional scale. The SMC responses to afforestation also vary across species. For example, significant SMC decrease are found for *Populus* spp. plantations (P < 0.05) and plantations of *Pinus sylvestris* var. *mongolica* (P < 0.05). Splitting the first meter of the soil profile into different depth intervals revealed that SMC declined significantly in shallow layers (0–30 cm) for *Populus* spp. and *Pinus sylvestris* var. *mongolica*. We also found that when SMC in the control exceeded a specific threshold, SMC for all five tree species considered tended to decrease, suggesting that the effects of afforestation on soil hydrology vary across different regions.

## Introduction

Afforestation and reforestation programs provide important mechanisms to enhance carbon sequestration capacity of the terrestrial ecosystem [1–3] and mitigate the increase of greenhouse gas emission. Such programs have been widely implemented in many regions, including America [4], South America [5], and China [6]. China, currently the largest carbon emitter of the world, is also known for containing more than 25% of the world’s planted forests [7]. Important afforestation projects in China include the Three-North Protective Forest Program, the River Protection Forest Program, and the Grain for Green Program [8]. Largely due to this afforestation effort, China’s forest coverage increased from 16.0% in the 1980s to 20.4% in 2009 [9], and forest carbon sequestration was about 1.65 Pg C during the same period, with a mean rate of 0.075 Pg C yr^-1^ [3].

However, afforestation does not come without any environmental cost, which is especially evident in arid and semi-arid regions [10–11]. One of the most striking issues associated with afforestation lies in its influence on hydrological cycles and modification of water resource balance [12–13]. In general, afforestation increases canopy interception and transpiration loss of water, and hence reduces river and stream runoff and water yield [14–15]. A global analysis of 504 annual catchment observations showed that afforestation dramatically decreased stream flow within a few years of forest planting [16]. In China’s Loess Plateau where afforestation programs like the Grain for Green Project have transformed 3.84 million hectares of farmland to forest plantations by the 2008 [17], which also bring out negative eco-hydrological effects like aggravated local water scarcity, even soil desiccation [10].

Soil moisture content (SMC) is one of the most important parameters determining the productivity and sustainability of terrestrial ecosystems [10,18,19]. Vegetation cover influences soil moisture through interception, transpiration and surface shading [20]. Because forest tends to have larger leaf area, surface roughness and deeper rooting system than grassland or cropland, vegetation restoration from cropland or grassland to forest could usually result in drastically reduced SMC restored [13], sometimes even to an extent of more than 35% [21], so that irrigation sometimes was performed to improve water availability [22]. Quantitative analyses of the influence on SMC by afforestation have been conducted in some scattered locations or regions [23–25], such as the Loess Plateau. However, these scattered results cannot be extrapolated to other forest plantations as such influence is highly heterogeneous in quantity across different regions [26].

Northeastern China is a key region for China’s Three-North Protective Forest Program, designed to hold back the expansion of the Gobi Desert. In this study, using pairwise sampling of 757 afforested and control plots, we first quantify the effect of large-scale afforestation on SMC across Northeastern China. Because traits like canopy structure and stomatal properties could vary across different tree species, the capacity of plantation forests in modifying hydrological cycle is also different across different forest types. Thus a second objective of this study is to compare the change of SMC across five tree species widely used in afforestation in Northeastern China. Finally, afforestation-caused changes in SMC could also be heterogeneous between shallow and deep soil layers [21]. Using soil profile data, we also demonstrate the differences of SMC and its change along soil depths. Overall, the results will provide a comprehensive understanding on how different choices of afforestation tree species may influence soil moisture across horizontal and vertical space, which is critical information for assessing the ongoing afforestation programs and optimizing the design of future ones.

## Materials and Methods

### Study Area

**Fig 1:**
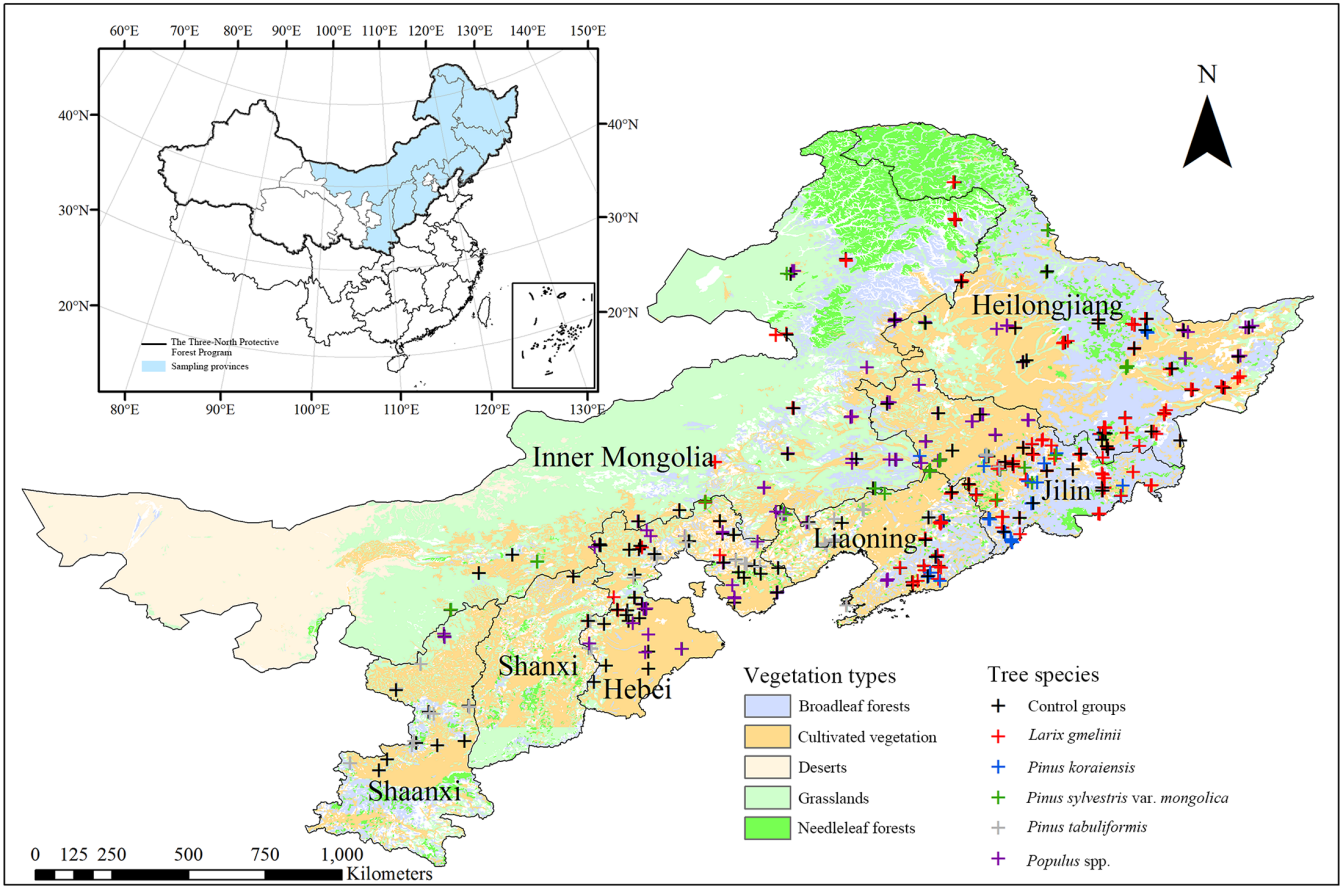
Map of the study area. The locations of sampling sites surveyed during 2012–2013 are marked with “+” symbols. The texts displayed refer to the name of provincial administrative unit, which are Inner Mongolia Autonomous Region, Shaanxi Province, Shanxi Province, Hebei Province, Liaoning Province, Jilin Province and Heilongjiang Province, respectively. Land cover map was adapted from the vegetation distribution map of China [28].

The sampling sites are located in Northeastern China (34.20–51.80°N, 106.81–133.31°E) covering the provinces of Heilongjiang, Jilin, Liaoning, Hebei, Shanxi, Shaanxi and the Inner Mongolia Autonomous Region (Fig 1). Over the study area, mean annual temperature ranges from -3 to 15°C, and the mean annual precipitation ranges from 355 to 1068 mm yr^-1^. Most of the sites are located in state-owned forest plantation farms and afforestation projects have been performed in a systematic way and well documented [27]. The administrative body of all involved forest farms gave permission for our research activities. The detailed information of forest farms are showed in S1 Table. Soil samples were obtained from plantation stands of five most commonly planted tree species (species groups): *Pinus koraiensis*, *Larix gmelinii*, *Pinus sylvestris* var. *mongolica*, *Pinus tabulaeformis* and *Populus* spp.

### Measurements

Gravimetric soil moisture measurements were made during the summers of 2012 and 2013 at a total of 172 field sites spreading across the study region (Fig 1). Each site contains three tree plantation plots of different ages and one pre-afforestation control plot. The plantation plots are from stands with common local species planted; and the control plots represent initial soil conditions before afforestation. At each sampling site, environmental attributes such as land-use type, aspect, slope degree, elevation, tree species and tree height were recorded. To ensure the consistent soil origin and climate, the distance between any two plantation plots within the same site is always between 50 m and 5 km, and the distance between each paired plantation-control plots is less than 2.5 km. At each plot, three soil pits were dug with a spade and soil samples were taken from depth of 0–5 cm, 5–10 cm, 10–20 cm, 20–30 cm, 30–60 cm, 60–100 cm using a standard 100-cm^3^ container. In total, 757 plots were sampled and 11,794 soil samples were collected. The soil samples were handled following standard procedures. Plant detritus were removed before weighing, and soils were sieved through a 2 mm mesh. All samples were weighed in the field, and then weighed again in the laboratory after drying at 105°C till constant weight.

### Data analysis

We denote the 0–5 cm, 5–10 cm, 10–20 cm, 20–30 cm, 30–60 cm, 60–100 cm depth intervals as d_1_, d_2_…d_6_ respectively, SMC (mass of soil water relative to dry-weight, %) of these depths as SMC_1_, SMC_2_,…SMC_6_, and top 1-m SMC as SMC_0-1m_.

Soil moisture contents are calculated as:

$$\text{SM}{\text{C}}_{\text{j}}=\frac{\left(\text{fresh weight}-\text{dry weight}\right)}{\text{dry weight}}\times 100\text{\% }(\text{j}=1,2\ldots 6)$$ (1)

The thickness-weighted method is used to calculate the SMC_0-1m_ (%), which is given by the equation

$$\text{SM}{\text{C}}_{0-1\text{m}}=\frac{\sum_{\text{i}=1}^{3}\sum_{\text{j}=1}^{6}\text{SM}{\text{C}}_{\text{ij}}\times {\text{w}}_{\text{ij}}}{\sum_{\text{i}=1}^{3}\sum_{\text{j}=1}^{6}{\text{w}}_{\text{ij}}}$$ (2)

where SMC_ij_ is SMC of the j^th^ layer in the i^th^ profile, w_ij_ is the weighting factor of the j^th^ layer in the i^th^ profile. Both SMC_a,0-1m_ and SMC_c,0-1m_ are calculated with Eq (2). Here the denotations *a* and *c* represent afforested and control plots, respectively. ΔSMC_0-1m_ refers to the difference between SMC_a,0-1m_ and SMC_c,0-1m_. ΔSMC_j_ refers to the difference between SMC_a,j_ and SMC_c,j_ (j = 1, 2, 3 … 6).

The mean and standard deviation (std) of change in top 1-m soil moisture content (ΔSMC_0-1m_) in each site are listed in S2 Table.

### Statistical methods

We performed Jarque-Bera test [29] and Brown-Forsythe's test [30] on the normality and homogeneity of variance, respectively. As it turns out the sample does not conform to normal distribution (P < 0.01), but met the variance homogeneity assumption (P > 0.05). So we use the non-parametric test in this study. A one-tailed paired Wilcoxon signed rank test was performed to detect whether SMC in afforested plots significantly decreased compared to SMC in control plots. The same tests were also performed on SMC of the six depth intervals. The relationship between the changes in top 1-m SMC after afforestation (ΔSMC_0-1m_) and SMC of top 1-m in control plots (SMC_c,0-1m_) was fitted using cubic polynomial. A one-tailed Mann Whitney U test was used to compare tree height differences between plots that SMCc,_0-1m_ are above and below the threshold are significantly higher than zero. Linear mixed model was fitted to evaluate tree height effects on ΔSMC_0-1m_ within each of the five tree species. Tree species was treated as fixed effects, while tree heights was treated as random effects. All of the comparisons were examined at a significance level of 0.05.

## Results

### Afforestation-induced change in SMC_0-1m_ across the study area

**Fig 2:**
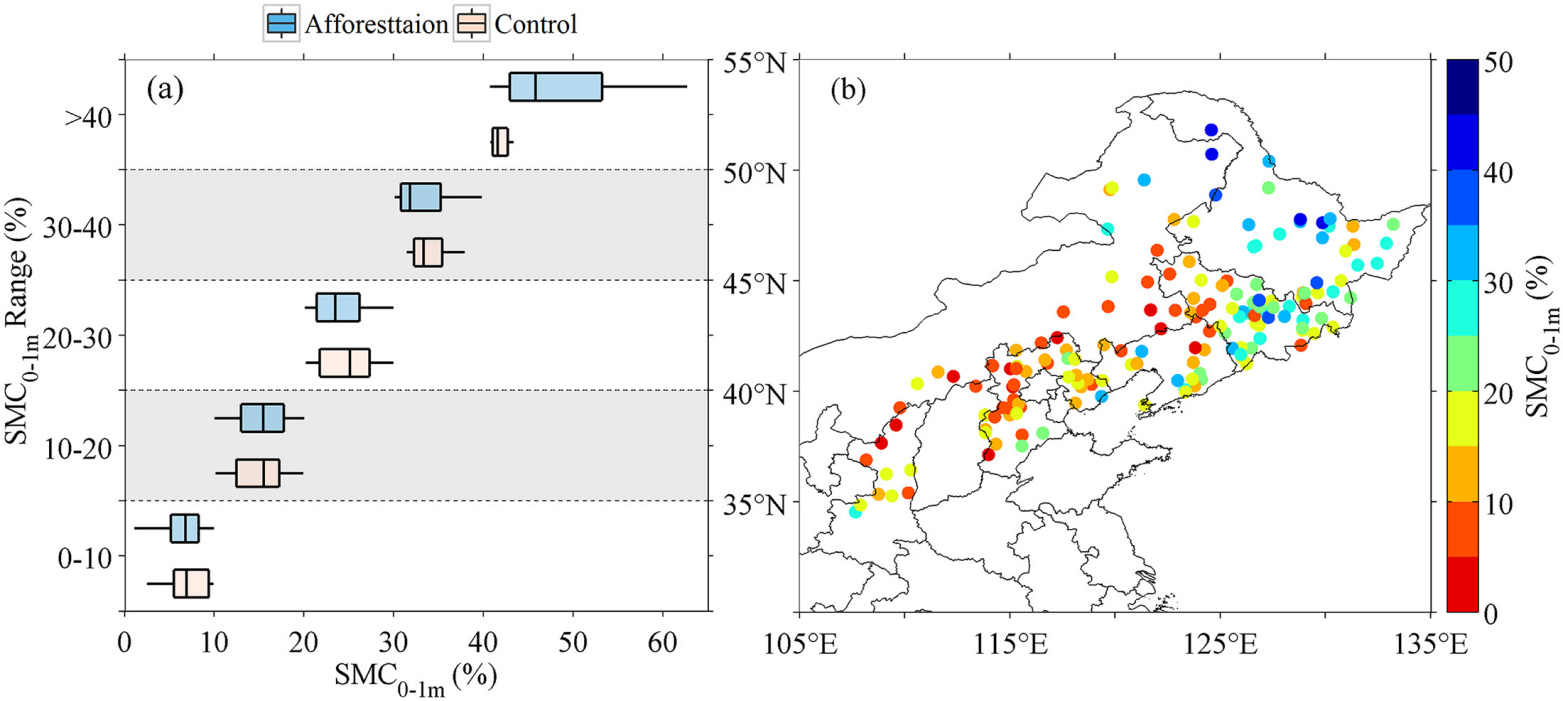
Moisture content of top 1-m soils (SMC_0-1m_). (a) Box-plots of SMC_0-1m_ in control and afforested plots. (b) Spatial distribution of SMC_0-1m_ in control plots. In box plots, the whiskers and boxes indicate the minimum, 25^th^ percentile, median, 75^th^ percentile and maximum.

**Fig 3:**
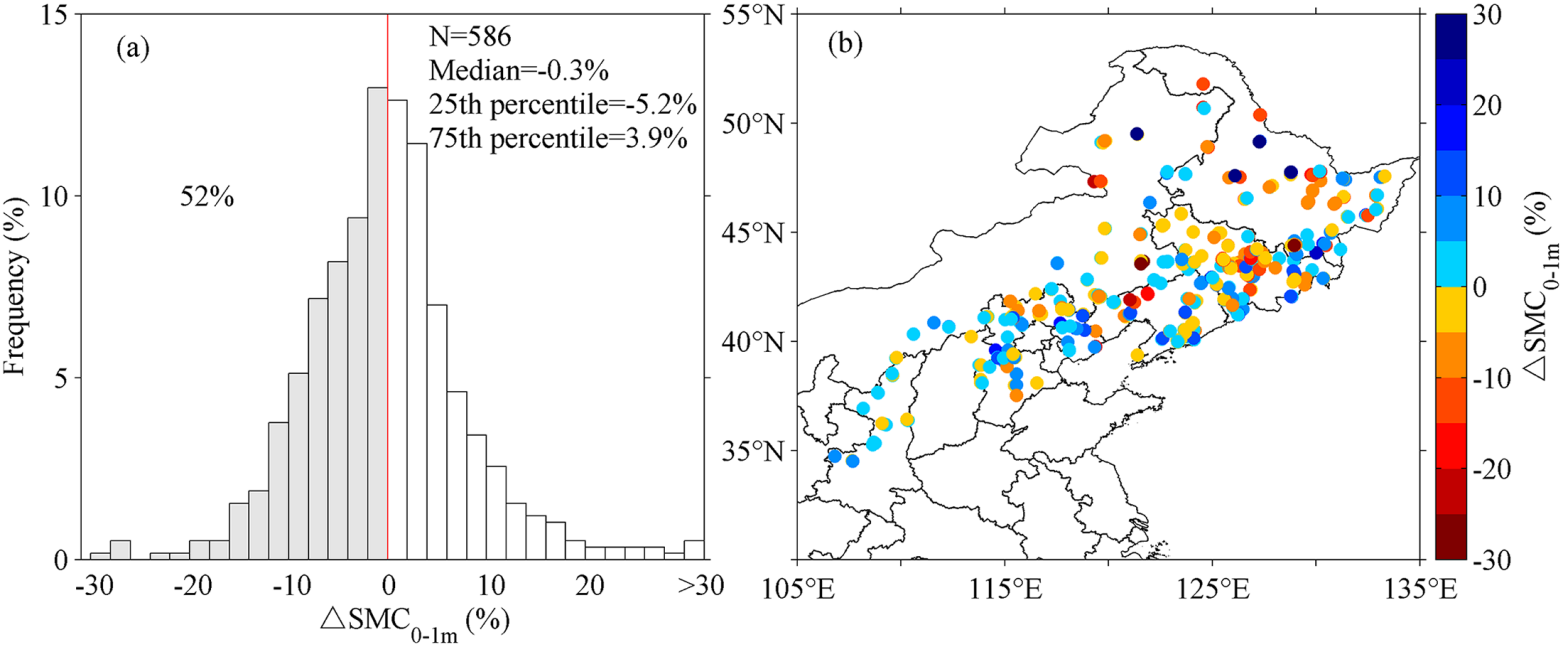
Changes of top 1-m soil moisture content (ΔSMC_0-1m_) in afforested plots (N = 586). (a) Frequency distribution of ΔSMC_0-1m_; (b) Spatial distribution of ΔSMC_0-1m._ In panel (a), the median, 25^th^ and 75^th^ percentile of ΔSMC_0-1m_ are displayed in texts. Negative ΔSMC_0-1m_ is found in 52% of the plots.

Fig 2a shows the box-plot of soil moisture content of top 1-m (SMC_0-1m_) in control and afforested plots. Soil moisture in control plots (SMC_c,0-1m_) has a wide range from 2.5% to 43.3% (Fig 2a), mostly due to a spatial gradient in SMC with the highest values in the northeast and lower values in the southwest. SMC_c,0-1m_ is mostly less than 20% in provinces of Hebei, Shanxi, Shaanxi, and Inner Mongolia, while it is relatively higher (>20%) in Heilongjiang, Jilin, and Liaoning (Fig 2b). On average, afforestation leads to marginally significant decrease in SMC_0-1m_ (P = 0.08), and the median of ΔSMC_0-1m_ is about -0.3%. ΔSMC_0-1m_ varies widely from -28.9% to 34.9% across different afforested plots. 52% of the afforested sites had a negative ΔSMC_0-1m_ (Fig 3a), mostly in Heilongjiang, Jilin, and Liaoning Provinces (Fig 3b) where SMC_c,0-1m_ is relatively high (Fig 2b).

**Fig 4:**
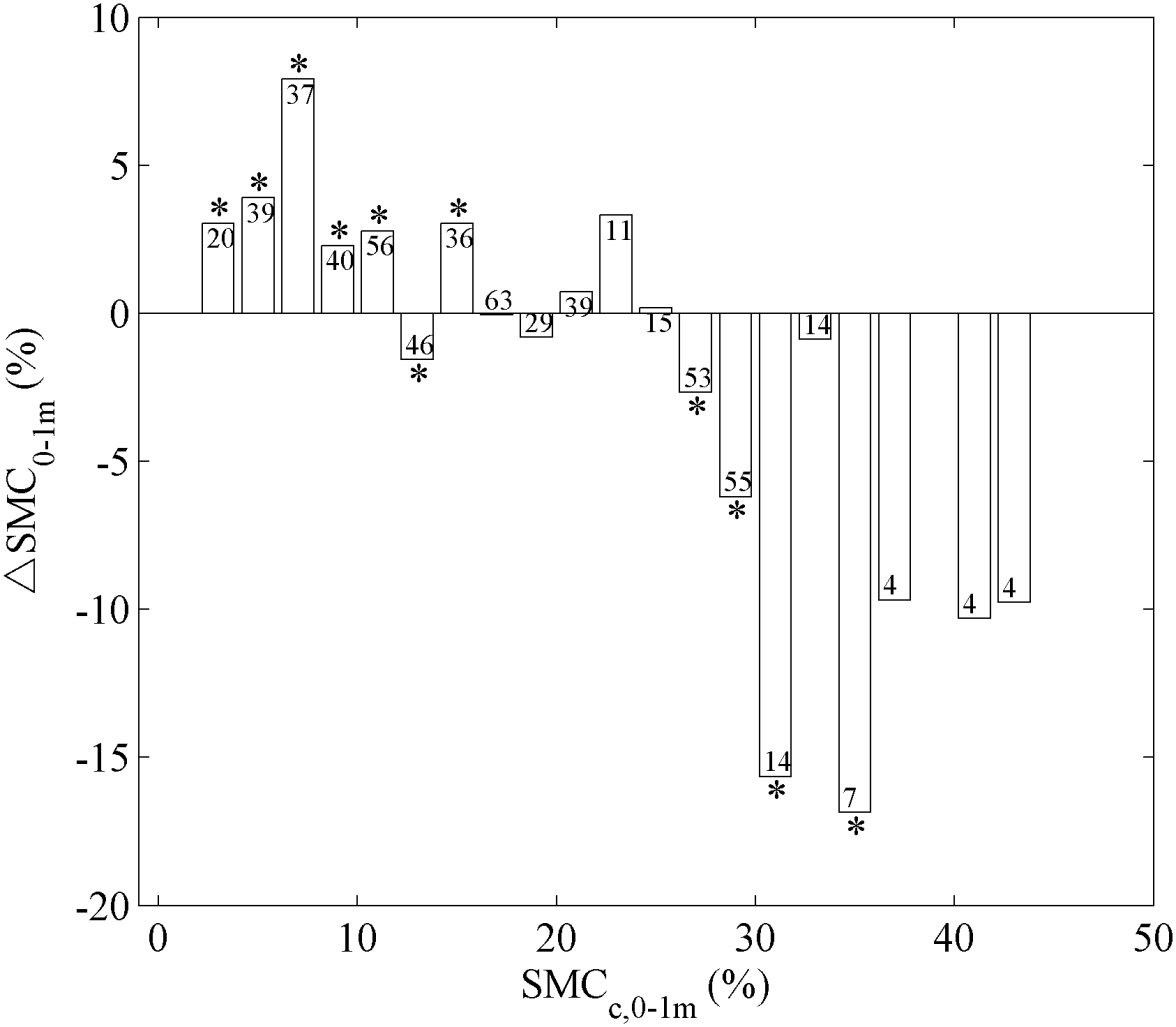
Relationships between the change of top 1-m soil moisture content (ΔSMC_0-1m_) and the top 1-m soil moisture content in control plots (SMC_c,0-1m_). SMC_c,0-1m_ was divided into bins by every 2% SMC_c,0-1m_. The numbers on top of the bars refer to the number of plots falling into each group. The asterisks (*) indicate significantly (P < 0.05) positive or negative non-zero ΔSMC_0-1m_ depended on the median of ΔSMC_0-1m_.

To further understand how pre-afforestation soil moisture content may affect afforestation-caused changes in SMC, we binned all the 586 afforested plots into 20 groups by their values of SMC_c,0-1m_ (Fig 4). The results suggest that at wet places where SMC_c,0-1m_ is larger than 25%, mean ΔSMC_0-1m_ is always negative. In relatively dry zones (SMC_c,0-1m_ is between 2% and 24%), both positive and negative values of ΔSMC_0-1m_ are found, with positive values more dominant (Fig 4).

### Afforestation-induced change in SMC_0-1m_ across different afforestation species

**Fig 5:**
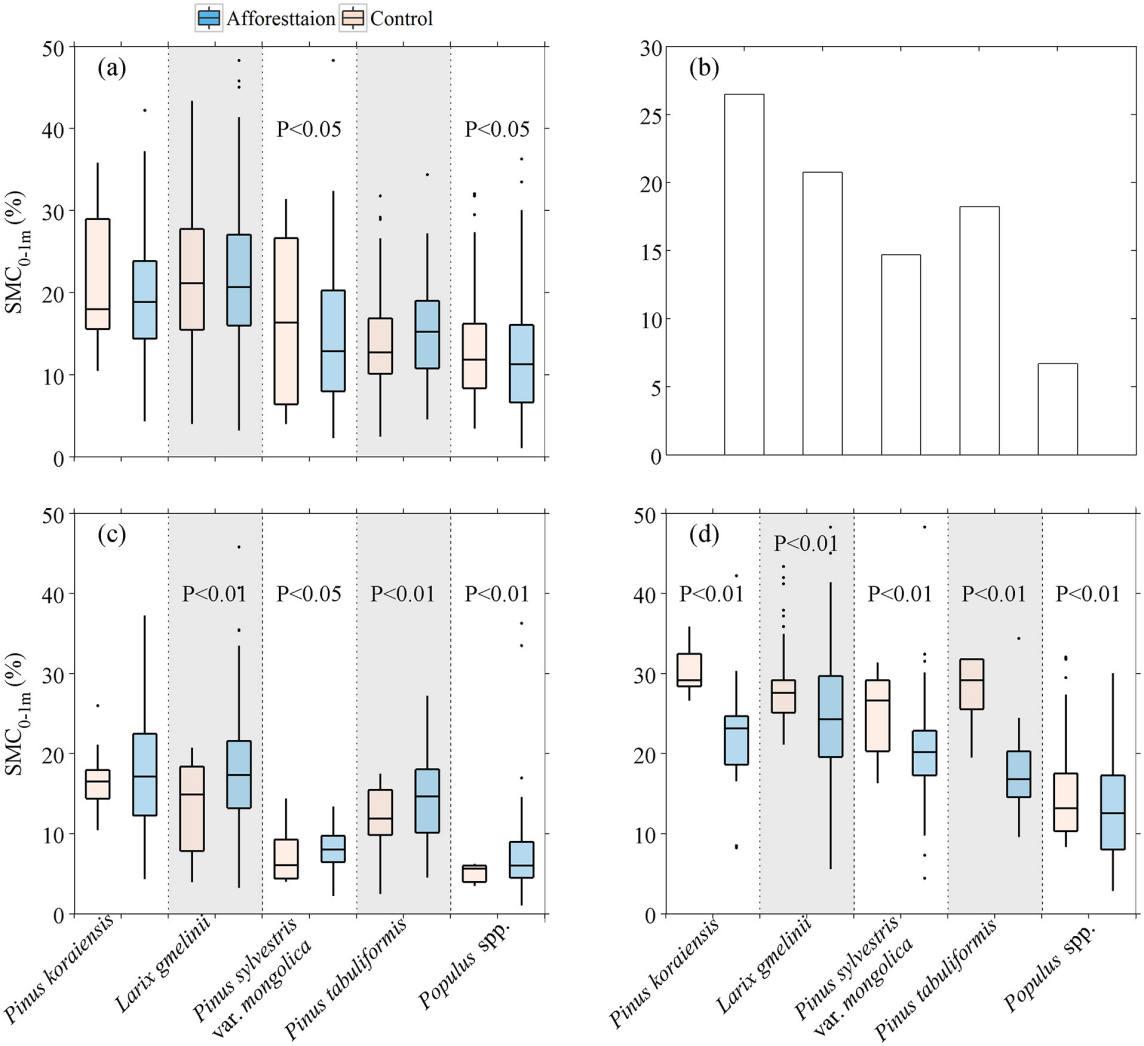
Soil moisture content of top 1-m (SMC_0-1m_) in control and afforested plots under different afforestation tree species. (a) SMC_0-1m_ in control and afforested plots. (b) The thresholds of top 1-m soil moisture content in control plots (SMC_c,0-1m_) when the change of top 1-m soil moisture content (ΔSMC_0-1m_) turns from positive to negative under different tree species, (c) SMC_0-1m_ in control and afforested plots when SMC_c,0-1m_ are below the threshold, and (d) above the threshold. In panel (a) and (d), the text P < 0.05 and P < 0.01 denote significantly and extremely significantly decrease in SMC_0-1m_ in afforestation plots compared to control plots. In panel (c), the text P < 0.05 and P < 0.01 denote significantly and extremely significantly increase in SMC_0-1m_ in afforestation plots compared to control plots. In box plots, the whiskers and boxes indicate the minimum, 25^th^ percentile, median, 75^th^ percentile and maximum.

Fig 5a shows the SMC_0-1m_ of control *vs*, afforested plots under different afforestation tree species. Significant decrease in SMC_0-1m_ are found for *Populus* spp. plantations (-0.68%, P < 0.05), and plantations of *Pinus sylvestris* var. *mongolica* (-0.50%, P < 0.05). And a marginally significant decrease occurred under plantations of *Pinus koraiensis* (P = 0.09). For the remaining other species, the change in SMC_0-1m_ is not significant (-0.35%—+1.66%, P > 0.1). For all species, significant decreases in SMC_0-1m_ (negative ΔSMC_0-1m_) are found when SMC_c,0-1m_ is high (S1 Fig), yet with species-specific threshold values of SMC_c,0-1m_ when ΔSMC_0-1m_ turns from positive to negative. The largest threshold of SMC_c,0-1m_ occurred in *Pinus koraiensis* plantations (around 26%) and the smallest one in *Populus* spp. (around 7%). The results suggest that *Pinus koraiensisi* plantations can reduce soil moisture content only in relatively wet regions, while *Populus* spp. plantations could lead to SMC_0-1m_ decline even in very dry regions.

We then divided the afforested plots into two categories, i.e., plots below and above the species-specific threshold of SMC_c,0-1m_ (Fig 5b). For the plots below the threshold of SMC_c,0-1m_, afforestation-induced increases in SMC_0-1m_ are statistically significant (P < 0.05, Fig 5c) for four of the five species, except for *Pinus koraiensis*. For plots with SMC_c,0-1m_ above the threshold, all five tree species show extremely significant negative ΔSMC_0-1m_ (P < 0.01, Fig 5d).

### Vertical distribution of afforestation-induced change in SMC among five tree species

**Fig 6:**
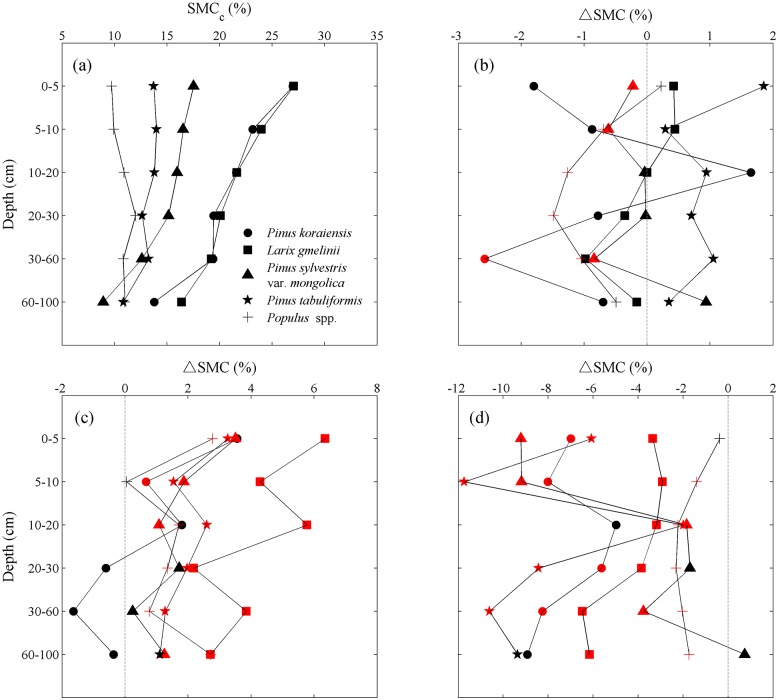
Vertical distribution of: (a) soil moisture content (SMC) in control plots, (b) the change of soil moisture content (ΔSMC), (c) ΔSMC when top 1-m soil moisture content in control plots (SMC_c,0-1m_) are below the threshold (threshold values are shown in Fig 5b), and (d) above the threshold under different tree species for six different soil depths (0–5 cm, 5–10 cm, 10–20 cm, 20–30 cm, 30–60 cm, 60–100 cm). In panel (b) and (d), the red points indicate a significant decline of SMC in afforestation plots compared to control plots (P < 0.05). In panel (c), the red points indicate a significant increase of SMC in afforestation plots compared to control plots.

We further explore afforestation-induced changes in soil moisture content (ΔSMC) along soil depths (Fig 6). Soil moisture content generally decreases with soil depths (Fig 6a), which is consistent with early findings [23]. However, ΔSMC does not show a consistent pattern along soil depth (Fig 6b). The largest magnitude of ΔSMC occurred in 10–60 cm soils of *Pinus koraiensis*, namely plantations, ranging from 1.6% to -2.6%. Non-significant ΔSMC are found in all soil layers of *Larix gmelinii* and *Pinus tabuliformis* plantations. Significant negative ΔSMC are also found in top 30-cm soils under plantations of *Populus* spp. and *Pinus* s*ylvestris* var. *mongolica*.

As we showed before, afforestation reduces soil moisture content in wet regions but may increases soil moisture in dry regions. Therefore, for each soil depth, we also identified the species-specific thresholds of SMC_c,0-1m_ (Fig 6c and 6d). As it turns out, when SMC_c,0-1m_ is below the thresholds, ΔSMC at most depth intervals are significantly positive for *Larix gmelinii*, *Pinus sylvestris* var. *mongolica*, *Populus* spp. and *Pinus tabuliformis*. The largest SMC increase occurs in *Larix gmelinii* (Fig 6c). On the contrary, when SMC_c,0-1m_ is above the thresholds, ΔSMC are negative over almost all soil depth intervals. The largest magnitude of ΔSMC is found in the 5–20 cm soils of *Pinus tabuliformis* plantations (-11.7% – -2.0%, Fig 6d).

## Discussion

### Impact of tree species on the SMC change after afforestation

Afforestation has been implemented in large areas of arid and semi-arid regions as an effective carbon sequestration and land management tool. There has been a growing knowledge that afforestation can alter hydrological cycle through modifying soil moisture [12] and changing stream flows [16,31,32]. For example, Yang et al. [13] evaluated the hydrological effect of vegetation restoration in Loess Plateau and found that soil moisture content decrease drastically after vegetation restoration. Compared to herbaceous vegetation, forest plantation was found to alter soil moisture even more by increasing rainfall interception and evapotranspiration [33], probably due to their larger leaf area [34] and deeper roots [35]. Consistent with previous studies, our results suggest that afforestation in Northeastern China marginally significantly reduces SMC (-0.3%, P = 0.08), although changes in SMC are not uniform across plantations with different tree species. For example, *Populus* spp. and *Pinus sylvestris* var. *mongolica* showed statistically significant SMC decline, while SMC change was not statistically significant under other tree species, including *Pinus koraiensis*, *Larix gmelinii* and *Pinus tabuliformis*. Such species-dependent impact on soil moisture has rarely been explored before (but sees Zhao [36] for plantations at Gobi).

The difference in canopy evapotranspiration across tree species may largely explain this inter-species difference in afforestation impacts on soil moisture. Many studies have found that *Populus* spp. plantations have a larger evapotranspiration capacity than most other tree species. For example, in a study over Horqin, among two studied tree species, the evapotranspiration of *Populus* spp. (558.1mm) is more than 30% larger than *Pinus sylvestris* var. *mongolica* plantations (405.0mm) during the growing season [37]. In Loess Plateau, the daily transpiration rate of *Populus* spp. (0.616 mmol/m^2^/s) is nearly twice more than *Pinus tabuliformis* (0.318 mmol/m^2^/s) [38]. Therefore, it is not surprising that afforestation with *Populus* spp. reduces SMC more than that with other species. Few studies have investigated evapotranspiration capacity of dominant tree species for afforestation in Northeastern China. Such information is required for comprehensive assessment of the ecosystem impacts by those afforestation projects.

Plant structural traits are also associated with regulation of transpiration [39], like canopy characteristics and root systems. Since tree height is a continuous variable, we fit a linear mixed model using tree species, tree height and their interaction as predictive terms to test whether canopy heights can influence the SMC change. As it turns out, tree height impacts on ΔSMC was not statistically significant (P = 0.40). Therefore, tree height differences may not be the main factor driving ΔSMC variations found in our study. We also appreciate other hypothesis concerning other forest traits, such as root depth. 98% roots of *Pinus sylvestris* var. *mongolica* distributed in 1.0m depth [40], while *Populus* spp. roots can extend to 2m [41], which leading to larger root distribution discrepancy between plantation and control plots. Therefore, *Populus* spp. may maintain their transpiration even water deficit appeared in shallow soil depth. Besides, *Populus* spp. has larger leaf area than *Pinus sylvestris* var. *mongolica* [42]. Thus higher canopy transpiration of *Populus* spp. would result in more soil moisture loss. However, due to data limitation, we cannot explore them quantitatively in this study.

The variation in growth rate across species may be another possible reason for the inter-species difference in the afforestation-caused SMC decrease [43]. Fast-growing species demands more water for its carbon assimilation [44], and would lead to a higher overall water use per rotation period [31]. For example, *Populus* spp. trees grows faster than other tree species in this study and also require more water during their life cycles [45]. This high water demand by fast-growing *Populus* spp. trees could create a larger difference in soil moisture content between pre-afforested and afforested plots than most other species. In fact, the different water consumption among tree species has already attracted attentions in afforestation species selection under different environment [25,46]. Given the high life-cycle water demand of *Populus* spp., currently extensive planting of *Populus* spp. in arid and semi-arid regions over Northeastern China may need to be reconsidered, and large-scale *Populus* spp. plantations would need to be assessed for their impacts on local water resources.

### Dependence of afforestation impacts on SMC in control plots

Soil moisture content of pre-afforested control plots can serve as the baseline SMC level of a region. Our results suggest that in addition to inter-species difference in afforestation’s impacts on soil moisture, baseline SMC also plays a critical role in determining the direction and amplitude of ΔSMC caused by afforestation. Significant soil moisture decrease (negative ΔSMC_0-1m_) is found when the baseline soil moisture (SMC_c,0-1m_) is larger than 25% (Fig 4). In wet places with higher water availability, trees tend to grow faster than in drier areas [47], which in return consumes more soil water [48]. Indeed, when we compared tree heights for plots with soil moisture above and below the threshold of SMC_c,0-1m_, three of the five species show at least significant (P < 0.05) higher trees in plots with higher SMC_c,0-1m_ (S2 Fig), supporting the hypothesis that higher soil moisture promotes tree growth and in turn leads to more depletion of soil moisture.

On the other hand, plants also develop physiological and morphological approaches in adaptation to drought. For instance, plants may respond to drought stress by closing their stomata [49], leading to reduced water consumption from the soil and slow-growing “small aged trees” in dry areas [50–51]. Also, it is worth to note that afforestation may even increase SMC in some arid areas when SMC_c,0-1m_ is between 2% and 24% (Fig 4). This positive change in SMC by afforestation in dry areas may be explained by irrigation and/or precipitation feedbacks. For example, irrigation has been widely employed for most plantation programs located in arid and semi-arid areas [22,52], which can offset or even exceed soil moisture deficit caused by increased canopy evapotranspiration. Also in those water-limited areas, there is a precipitation feedback whereby increased evapotranspiration tends to favor more precipitation [53–54]. It is suggested that even in those dry regions, evapotranspiration of forested lands is still greater than that of non-forested lands [55]. The water vapor from forested lands may modify regional rainfall patterns and trigger more precipitation, and thus increase soil moisture content.

The hypotheses of irrigation and precipitation feedbacks proposed here are currently difficult to test because of the lack of data for the spatial extent of irrigation practice and the complexity in modeling regional precipitation feedback to afforestation. However, our results highlight that complicated influences of afforestation on local hydrological cycle. Afforestation programs often face the challenge of the competing demands between water resources and carbon sequestration, which requires careful selection of tree species and delicately balancing both objects. Current understanding on how to satisfy both water and carbon goals in afforestation programs is still limited. Compared with pair-wise catchment study (e.g. Huang et al [56]), our paired-plot sampling approach is subject to more sources of uncertainties. In particular, although the pair-sampling is always taken at the same day, the magnitude of the soil moisture difference between control-plots and afforestation-plots may be affected by seasonal variations of climate. However, this limitation did not affect our qualitative inference that afforestation has led to marginally significant change on regional hydrological cycle. Improving Earth system modeling and more soil moisture data with continuous monitoring should become the research priorities in future in order to better quantify the afforestation impacts.

## Supporting Information

S1 Fig Relationships between change of top 1m soil moisture content (ΔSMC_0-1m_) and top 1m soil moisture content in control plots (SMC_c,0-1m_) for five tree species. (a) *Pinus koraiensis*, (b) *Larix gmelinii*, (c) *Pinus sylvestris* var. *mongolica*, (d) *Pinus tabuliformis*, (e) *Populus* spp.. SMC_c,0-1m_ was divided into 25 bins. The numbers in the top right are the total number of afforested plots within plantations of specific tree species. The asterisks (*) indicate significantly (P < 0.05) positive or negative non-zero ΔSMC_0-1m_ depended on the median of ΔSMC_0-1m_.
(TIF)

S2 Fig Tree height differences between plots that top 1m soil moisture content in control plots (SMC_c,0-1m_) are above and below the threshold for five tree species. Error bars show confidence interval of tree height difference. The asterisks (*) denote that tree height differences between plots that SMC_c,0-1m_ are above and below the threshold are significantly higher than zero, * P < 0.05, ** P < 0.01.
(TIF)

S1 Table Sample sites information in our experiment. The longitude, latitude and name of forest farms are provided as below.
(DOCX)

S2 Table Sample data in our experiment. The mean and standard deviation (std) of change in top 1-m soil moisture content (ΔSMC_0-1m_) in each site.
(DOCX)
