# Supplementary figures and images for: The Effect of Afforestation on Soil Moisture Content in Northeastern China
Source: PLoS One. 2016 Aug 11;11(8):e0160776. doi: 10.1371/journal.pone.0160776 (PMC4981471; doi:10.1371/journal.pone.0160776)

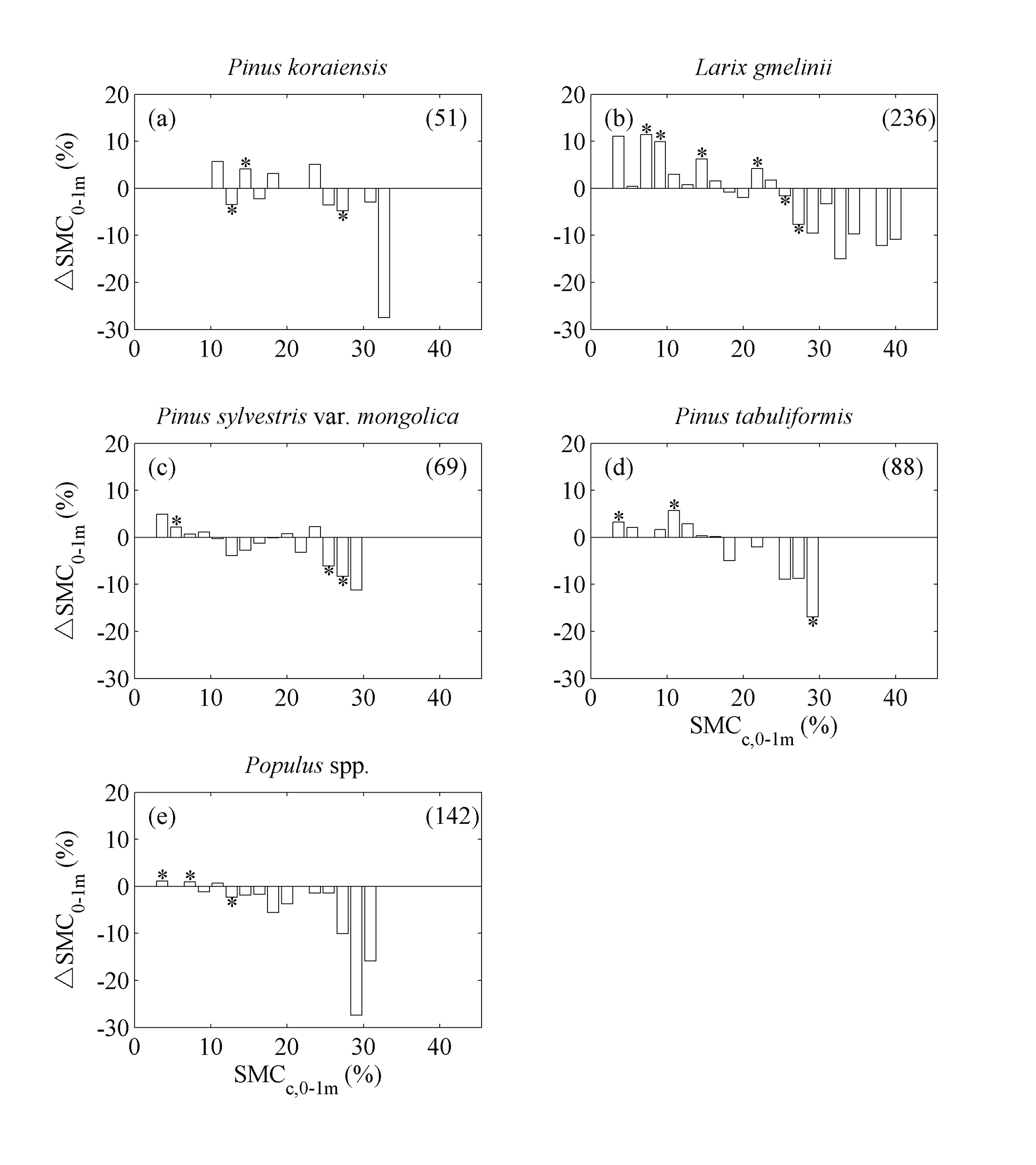

Supplement: S1 Fig — (a) Pinus koraiensis, (b) Larix gmelinii, (c) Pinus sylvestris var. mongolica, (d) Pinus tabuliformis, (e) Populus spp.. SMCc,0-1m was divided into 25 bins. The numbers in the top right are the total number of afforested plots within plantations of specific tree species. The asterisks (*) indicate significantly (P < 0.05) positive or negative non-zero ΔSMC0-1m depended on the median of ΔSMC0-1m. (TIF) [file pone.0160776.s001.tif]

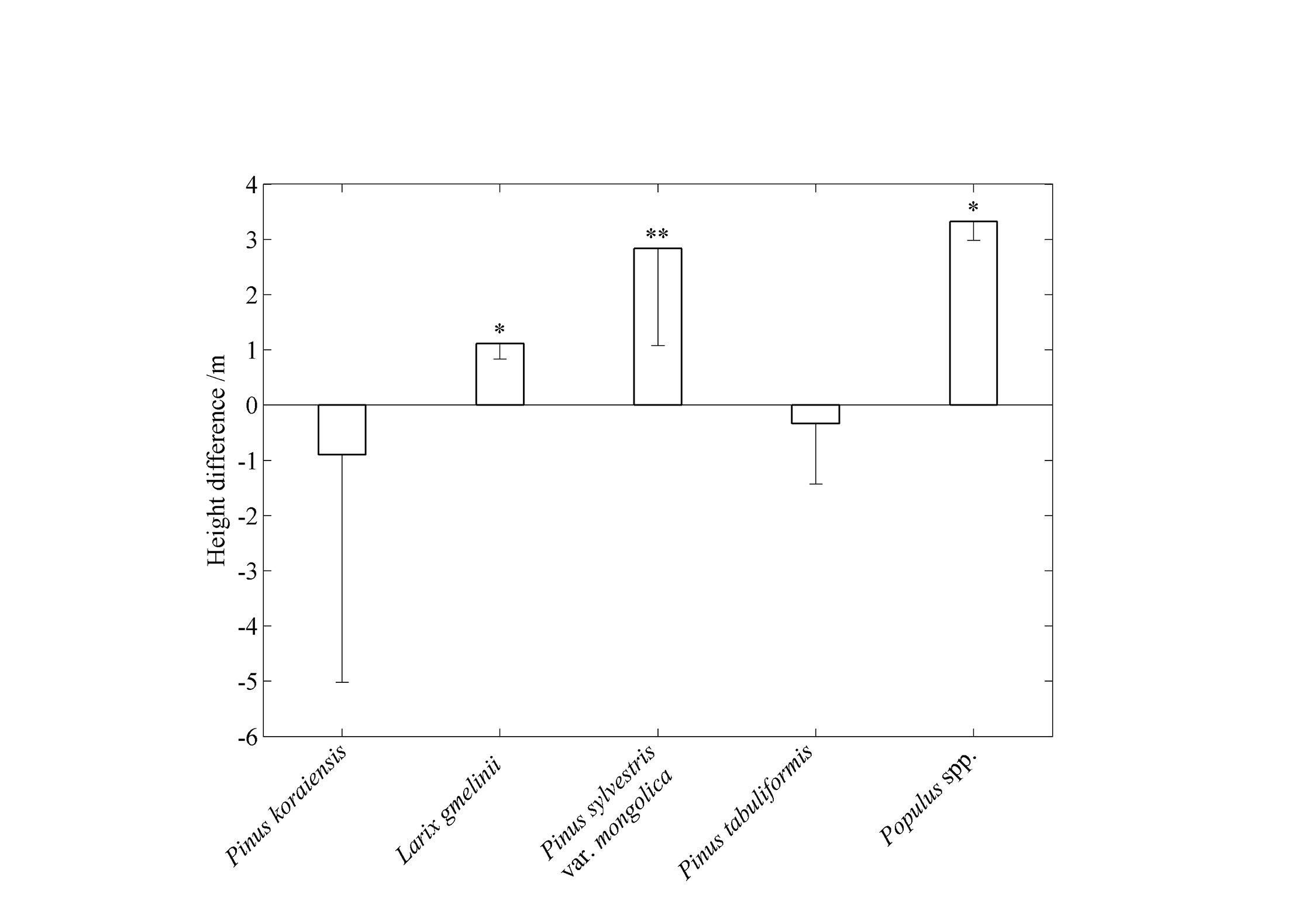

Supplement: S2 Fig — Error bars show confidence interval of tree height difference. The asterisks (*) denote that tree height differences between plots that SMCc,0-1m are above and below the threshold are significantly higher than zero, * P < 0.05, ** P < 0.01. (TIF) [file pone.0160776.s002.tif]
