# Supplementary material for: The Effect of Afforestation on Soil Moisture Content in Northeastern China
Source: PLoS One. 2016 Aug 11;11(8):e0160776. doi: 10.1371/journal.pone.0160776 (PMC4981471; doi:10.1371/journal.pone.0160776)
Supplement: S1 Table — The longitude, latitude and name of forest farms are provided as below. (DOCX) [file pone.0160776.s003.docx]

**S1 Table. Sample sites information in our experiment.** The longitude, latitude and name of forest farms are provided as below.

| No. | Province | Forest farm name | Longitude | Latitude |
| --- | --- | --- | --- | --- |
| 1 | Hebei | Xinzhuang Forest farm | 113.90 | 38.10 |
| 2 | Hebei | Beizhixiang Forest farm | 113.84 | 38.25 |
| 3 | Hebei | Yangze Forest farm | 114.33 | 37.60 |
| 4 | Hebei | Luluo Forest farm | 113.98 | 37.13 |
| 5 | Hebei | Shenxingzhen Forest farm | 115.22 | 39.00 |
| 6 | Hebei | Wanmu Forest farm | 115.39 | 39.42 |
| 7 | Hebei | Xiexiang Forest farm | 115.44 | 39.27 |
| 8 | Hebei | Qiaojiahe Forest farm | 114.95 | 39.23 |
| 9 | Hebei | Baishishan Forest farm | 114.68 | 39.23 |
| 10 | Hebei | Zhaogezhuang Forest farm | 115.16 | 39.60 |
| 11 | Hebei | Hekou Forest farm | 114.99 | 38.91 |
| 12 | Hebei | Wanglinkou Forest farm | 114.27 | 38.83 |
| 13 | Hebei | Luanzhuang Forest farm | 115.18 | 40.28 |
| 14 | Hebei | Air grassland | 114.56 | 39.61 |
| 15 | Hebei | Nanhaozhan Forest farm | 114.00 | 41.08 |
| 16 | Hebei | Baiqi Forest farm | 115.30 | 41.02 |
| 17 | Hebei | Shizuizi Forest farm | 115.01 | 41.00 |
| 18 | Hebei | Diao’e Forest farm | 115.83 | 40.79 |
| 19 | Hebei | Tuchengzhen Forest farm | 115.64 | 41.40 |
| 20 | Hebei | Nanguan Forest farm | 116.77 | 41.26 |
| 21 | Hebei | Lijiaying Forest farm | 117.78 | 40.63 |
| 22 | Hebei | Qijia Forest farm | 118.07 | 41.45 |
| 23 | Hebei | Tangtougou Forest farm | 117.75 | 41.46 |
| 24 | Hebei | Wolongzhen Forest farm | 118.75 | 41.17 |
| 25 | Hebei | Xiabancheng Forest farm | 118.14 | 40.69 |
| 26 | Hebei | Kuanchengzhen Forest farm | 118.45 | 40.58 |
| 27 | Hebei | Guoyuanxiang Forest farm | 118.10 | 37.62 |
| 28 | Hebei | Qianxi Forest farm | 118.21 | 40.37 |
| 29 | Hebei | Qianxi Forest farm | 118.38 | 40.19 |
| 30 | Hebei | Funing Forest farm | 119.34 | 39.74 |
| 31 | Hebei | Qinglong Forest farm | 118.85 | 40.51 |
| 32 | Hebei | Qinglong Forest farm | 119.38 | 40.47 |
| 33 | Hebei | Qinglong Forest farm | 118.87 | 40.29 |
| 34 | Hebei | Jixian Forest farm | 115.57 | 37.52 |
| 35 | Hebei | Shenzhoushi Forest farm | 115.57 | 38.01 |
| 36 | Hebei | Botoushi Forest farm | 116.56 | 38.10 |
| 37 | Heilongjiang | Wumasuo Forest farm | 128.78 | 47.76 |
| 38 | Heilongjiang | Shuangfeng Forest farm | 130.16 | 47.79 |
| 39 | Heilongjiang | Xiahua Forest farm | 130.87 | 46.30 |
| 40 | Heilongjiang | Hongxing Forest farm | 128.95 | 44.40 |
| 41 | Heilongjiang | Yong’anxiang Forest farm | 126.68 | 46.56 |
| 42 | Heilongjiang | Baiquanxian Forest farm | 126.10 | 47.59 |
| 43 | Heilongjiang | Xingshan Forest farm | 127.74 | 47.07 |
| 44 | Heilongjiang | Baishan Forest farm | 127.76 | 47.07 |
| 45 | Heilongjiang | Hongqi Forest farm | 130.23 | 47.37 |
| 46 | Heilongjiang | Junchuan Forest farm | 131.26 | 47.45 |
| 47 | Heilongjiang | Heijinhe Forest farm | 129.84 | 46.90 |
| 48 | Heilongjiang | Sibao Forest farm | 131.32 | 46.63 |
| 49 | Heilongjiang | Bawusan Forest farm | 131.34 | 46.62 |
| 50 | Heilongjiang | Qianjin Forest farm | 133.31 | 47.58 |
| 51 | Heilongjiang | Zhushan Forest farm | 129.60 | 46.38 |
| 52 | Heilongjiang | Bawusi Forest farm | 132.87 | 46.04 |
| 53 | Heilongjiang | Bawuling Forest farm | 132.50 | 45.76 |
| 54 | Heilongjiang | Bawuwu Forest farm | 131.56 | 45.71 |
| 55 | Heilongjiang | Qianjin Forest farm | 130.77 | 45.10 |
| 56 | Heilongjiang | Shanshi Forest farm | 128.95 | 44.61 |
| 57 | Heilongjiang | Toudao Forest farm | 129.57 | 44.89 |
| 58 | Heilongjiang | Donghe Forest farm | 129.61 | 44.45 |
| 59 | Heilongjiang | Hubei Forest farm | 129.06 | 43.98 |
| 60 | Heilongjiang | Nantianmen Forest farm | 131.19 | 44.21 |
| 61 | Heilongjiang | Hongyanjingyingsuo Forest farm | 130.41 | 44.47 |
| 62 | Jilin | Changren Forest farm | 128.96 | 42.82 |
| 63 | Jilin | Guangping Forest farm | 128.97 | 42.84 |
| 64 | Jilin | Shimen Forest farm | 128.82 | 42.07 |
| 65 | Jilin | Changxing Forest farm | 128.95 | 43.10 |
| 66 | Jilin | Beishan park Forest farm | 130.33 | 42.89 |
| 67 | Jilin | Longjing Forest farm | 129.50 | 42.89 |
| 68 | Jilin | Dongguangzhen Forest farm | 129.80 | 43.29 |
| 69 | Jilin | Helong Forest farm | 129.45 | 42.60 |
| 70 | Jilin | Xinxiangcun Forest farm | 128.03 | 43.37 |
| 71 | Jilin | Dunhua Forest farm | 128.23 | 43.79 |
| 72 | Jilin | Xiaocheng Forest farm | 127.15 | 44.25 |
| 73 | Jilin | Shiyan Forest farm | 123.54 | 43.75 |
| 74 | Jilin | Dabei Forest farm | 126.80 | 44.17 |
| 75 | Jilin | Shiyan Forest farm | 125.51 | 43.77 |
| 76 | Jilin | Nangangzi Forest farm | 127.55 | 43.83 |
| 77 | Jilin | Shaoguoxiang Forest farm | 125.89 | 43.35 |
| 78 | Jilin | Tiannan Forest farm | 126.85 | 43.80 |
| 79 | Jilin | Buhai Forest farm | 125.76 | 44.38 |
| 80 | Jilin | Hengdaozi Forest farm | 127.26 | 43.31 |
| 81 | Jilin | Halahai Forest farm | 125.07 | 44.77 |
| 82 | Jilin | Songjiang Forest farm | 127.19 | 43.54 |
| 83 | Jilin | Chengfa Forest farm | 126.71 | 44.81 |
| 84 | Jilin | Pingchuan Forest farm | 127.41 | 44.06 |
| 85 | Jilin | Shenyangxiang Forest farm | 124.14 | 43.65 |
| 86 | Jilin | Wulihe Forest farm | 126.62 | 43.42 |
| 87 | Jilin | Shiyan Forest farm | 123.58 | 43.55 |
| 88 | Jilin | Shuanghezhen Forest farm | 126.30 | 43.45 |
| 89 | Jilin | Gongzhuling Forest farm | 124.49 | 43.92 |
| 90 | Jilin | Jiangbei Forest farm | 126.57 | 44.01 |
| 91 | Jilin | Zengshengxiang Forest farm | 125.38 | 44.98 |
| 92 | Jilin | Dagangzi Forest farm | 126.05 | 43.58 |
| 93 | Jilin | Qianqihaoxiang Forest farm | 123.72 | 44.19 |
| 94 | Jilin | Xiyang Forest farm | 126.28 | 43.61 |
| 95 | Jilin | Qian’anzhen Forest farm | 124.06 | 45.01 |
| 96 | Jilin | Qingshui Forest farm | 126.67 | 43.06 |
| 97 | Jilin | Heiyupao Forest farm | 123.51 | 45.84 |
| 98 | Jilin | Daboji Forest farm | 126.83 | 42.97 |
| 99 | Jilin | Taonan Forest farm | 122.66 | 45.33 |
| 100 | Jilin | Baiquan Forest farm | 125.00 | 42.91 |
| 101 | Jilin | Yangmu Forest farm | 125.20 | 42.63 |
| 102 | Jilin | Xingling Forest farm | 125.77 | 42.44 |
| 103 | Jilin | Jinchang Ski field | 125.96 | 41.65 |
| 104 | Jilin | Sanpeng Forest farm | 125.56 | 41.92 |
| 105 | Jilin | Guanghua Forest farm | 125.98 | 41.96 |
| 106 | Jilin | Daqinggou Forest farm | 126.24 | 41.23 |
| 107 | Jilin | Toudao Forest farm | 126.05 | 41.51 |
| 108 | Jilin | Badaojiang Forest farm | 126.46 | 41.94 |
| 109 | Jilin | Zhenjiao Forest farm | 126.81 | 42.36 |
| 110 | Liao ning | Qinu’erhushan Forest farm | 120.22 | 41.80 |
| 111 | Liao ning | Heishui Forest farm | 119.52 | 42.05 |
| 112 | Liao ning | Qingshan Forest farm | 121.03 | 41.90 |
| 113 | Liao ning | Zhoujiadian Forest farm | 121.89 | 42.17 |
| 114 | Liao ning | Zhanggutai Forest farm | 122.51 | 42.65 |
| 115 | Liao ning | Cuiyanzhen Forest farm | 121.06 | 41.31 |
| 116 | Liao ning | Jianyi Forest farm | 122.96 | 40.47 |
| 117 | Liao ning | Wanfu Forest farm | 122.61 | 40.14 |
| 118 | Liao ning | Wafangdian Forest farm | 121.38 | 39.38 |
| 119 | Liao ning | Anzishanzhen Forest farm | 123.35 | 39.98 |
| 120 | Liao ning | Xiaoxigoucun Forest farm | 121.02 | 41.26 |
| 121 | Liao ning | Qingliangshan Forest farm | 123.66 | 40.54 |
| 122 | Liao ning | Dongfeng Forest farm | 123.69 | 40.40 |
| 123 | Liao ning | Youyanxian Forest farm | 123.48 | 40.09 |
| 124 | Liao ning | Fengchengshi Forest farm | 124.09 | 40.15 |
| 125 | Liao ning | Tongyuanbao Forest farm | 124.01 | 40.79 |
| 126 | Liao ning | Baoshan Forest farm | 123.86 | 40.33 |
| 127 | Liao ning | Fengshan Forest farm | 124.10 | 40.53 |
| 128 | Liao ning | Caitun Forest farm | 123.70 | 41.34 |
| 129 | Liao ning | Huiyuan Forest farm | 123.88 | 41.96 |
| 130 | Liao ning | Wendao Forest farm | 124.12 | 41.80 |
| 131 | Liao ning | Gaojiadian Forest farm | 124.45 | 42.65 |
| 132 | Liao ning | Fujiadian Forest farm | 123.83 | 43.29 |
| 133 | Neimenggu | Daqinggou Forest farm | 122.19 | 42.81 |
| 134 | Neimenggu | Dongfeng Forest farm | 121.55 | 43.55 |
| 135 | Neimenggu | Tu’erji Forest farm | 122.82 | 43.65 |
| 136 | Neimenggu | Hanshan Forest farm | 119.81 | 45.17 |
| 137 | Neimenggu | Cha’ersen Forest farm | 121.99 | 46.36 |
| 138 | Neimenggu | Dula’er Forest farm | 119.62 | 47.35 |
| 139 | Neimenggu | Haila’er Forest farm | 119.80 | 49.19 |
| 140 | Neimenggu | Center miaopu Forest farm | 119.75 | 49.10 |
| 141 | Neimenggu | 59 guanhu station | 121.37 | 49.50 |
| 142 | Neimenggu | Chengjisihan Forest farm | 122.83 | 47.76 |
| 143 | Neimenggu | Guli Forest farm | 124.60 | 50.68 |
| 144 | Neimenggu | Dawusu Forest farm | 124.54 | 51.79 |
| 145 | Neimenggu | Dawo’erzuqingnian Forest farm | 123.69 | 47.69 |
| 146 | Neimenggu | Fuyuan Forest farm | 124.75 | 48.91 |
| 147 | Neimenggu | Jiangfang Forest farm | 124.31 | 50.38 |
| 148 | Neimenggu | Chenqing Forest farm | 127.26 | 49.14 |
| 149 | Neimenggu | Yihetala Forest farm | 121.51 | 44.90 |
| 150 | Neimenggu | Yajishan Forest farm | 118.97 | 42.84 |
| 151 | Neimenggu | Lindong Forest farm | 119.67 | 43.83 |
| 152 | Neimenggu | Huanggangliang Forest farm | 117.53 | 43.57 |
| 153 | Neimenggu | Gongbaolage state-own Forest farm | 115.27 | 41.84 |
| 154 | Neimenggu | Duolun Forest farm | 116.48 | 42.18 |
| 155 | Neimenggu | Longtoushan Forest farm | 117.53 | 43.58 |
| 156 | Neimenggu | Saihanbajixie Forest farm | 117.25 | 42.41 |
| 157 | Neimenggu | Manhanshan Forest farm | 112.30 | 40.66 |
| 158 | Neimenggu | Wusutu Forest farm | 111.58 | 40.86 |
| 159 | Neimenggu | Huanghe Forest farm | 110.59 | 40.33 |
| 160 | Neimenggu | Xinjiezhisha station | 109.78 | 39.25 |
| 161 | Shanxi | Shiyan Forest farm | 113.37 | 40.21 |
| 162 | Shaanxi | Xiaojihan Forest farm | 109.57 | 38.54 |
| 163 | Shaanxi | Shashimao Forest farm | 108.88 | 37.65 |
| 164 | Shaanxi | Baishuling Forest farm | 110.28 | 36.43 |
| 165 | Shaanxi | Shenglishan Forest farm | 108.17 | 36.93 |
| 166 | Shaanxi | Fucun Forest farm | 109.10 | 36.25 |
| 167 | Shaanxi | Malan Forest farm | 108.76 | 35.33 |
| 168 | Shaanxi | Ximiaotou Forest farm | 107.93 | 34.87 |
| 169 | Shaanxi | Badu Forest farm | 106.82 | 34.75 |
| 170 | Shaanxi | Jueshan Forest farm | 107.67 | 34.51 |
| 171 | Shaanxi | Xinzhuo Forest farm | 109.37 | 35.28 |
| 172 | Shaanxi | Huangpu Forest farm | 110.16 | 35.41 |
