# Supplementary material for: The Effect of Afforestation on Soil Moisture Content in Northeastern China
Source: PLoS One. 2016 Aug 11;11(8):e0160776. doi: 10.1371/journal.pone.0160776 (PMC4981471; doi:10.1371/journal.pone.0160776)
Supplement: S2 Table — The mean and standard deviation (std) of change in top 1-m soil moisture content (ΔSMC0-1m) in each site. (DOCX) [file pone.0160776.s004.docx]

**S2 Table. Sample data in our experiment.** The mean and standard deviation (std) of change in top 1-m soil moisture content (△SMC_0-1m_) in each site. (There are 9 sites that only control plots data are available, so they are not listed here.)

| No. | Site No. | △SMC_0-1m_  (mean ± std, %) |
| --- | --- | --- |
| 1 | HB01 | -0.07±4.52 |
| 2 | HB02 | -4.02±4.3 |
| 3 | HB08 | 3.36±3.04 |
| 4 | HB09 | 1.16±4.42 |
| 5 | HB10 | 3.89±0 |
| 6 | HB11 | 14.01±11.91 |
| 7 | HB12 | 1.98±5.41 |
| 8 | HB13 | -0.22±8.51 |
| 9 | HB14 | -7.03±7.26 |
| 10 | HB15 | 2.88±1.71 |
| 11 | HB16 | 15.85±0 |
| 12 | HB17 | -1.11±2.99 |
| 13 | HB20 | 0.86±7.03 |
| 14 | HB21 | 1.86±1.44 |
| 15 | HB22 | 5.37±3.05 |
| 16 | HB23 | -6.92±2.97 |
| 17 | HB24 | -1.87±2.48 |
| 18 | HB28 | 1.81±0.71 |
| 19 | HB29 | 4.68±6.65 |
| 20 | HB30 | -4.57±3.63 |
| 21 | HB31 | 3.17±10.72 |
| 22 | HB32 | 9.14±9.79 |
| 23 | HB33 | 8.54±3.5 |
| 24 | HB34 | 2.95±3.47 |
| 25 | HB37 | -4.39±16.18 |
| 26 | HB38 | 4.76±5.94 |
| 27 | HB39 | -3.89±4.36 |
| 28 | HB41 | -0.57±8.55 |
| 29 | HB42 | 3.89±8.16 |
| 30 | HB43 | -0.2±3.26 |
| 31 | HL01 | 18.43±18.74 |
| 32 | HL02 | -6.68±15.81 |
| 33 | HL03 | -7.06±2.36 |
| 34 | HL04 | -6.07±12.15 |
| 35 | HL05 | -2.96±5.74 |
| 36 | HL06 | 6.28±20.88 |
| 37 | HL08 | -2.63±2.77 |
| 38 | HL09 | -8.21±9.09 |
| 39 | HL10 | -4.78±2.33 |
| 40 | HL11 | 2.67±3.58 |
| 41 | HL12 | 3.09±19.22 |
| 42 | HL13 | -9.39±6.12 |
| 43 | HL14 | -1.71±6.06 |
| 44 | HL15 | 0.41±4.46 |
| 45 | HL16 | -3.82±4.89 |
| 46 | HL17 | 2.01±4.05 |
| 47 | HL18 | 3.26±11.6 |
| 48 | HL19 | 0.24±4.3 |
| 49 | HL20 | 3.81±4.51 |
| 50 | HL21 | 4.08±4.54 |
| 51 | HL22 | -10.96±10.39 |
| 52 | HL23 | 7.75±4.6 |
| 53 | HL24 | 11.06±6.7 |
| 54 | HL25 | 1.34±4.62 |
| 55 | HL26 | 2.94±11.96 |
| 56 | JL01 | -5.22±1.63 |
| 57 | JL02 | 18.44±4.5 |
| 58 | JL03 | 7.05±3.91 |
| 59 | JL04 | 9.07±5.37 |
| 60 | JL05 | 0.39±5.49 |
| 61 | JL06 | -5.93±1.25 |
| 62 | JL07 | 6.32±3.84 |
| 63 | JL08 | -4.3±2.63 |
| 64 | JL09 | -6.17±2.93 |
| 65 | JL10 | 3.09±1.03 |
| 66 | JL11 | 3.36±6.16 |
| 67 | JL12 | -7.51±5.92 |
| 68 | JL13 | -10.66±0.95 |
| 69 | JL14 | -9.25±0.69 |
| 70 | JL15 | -7.69±3.9 |
| 71 | JL16 | -2.3±5.4 |
| 72 | JL17 | -0.97±9.69 |
| 73 | JL18 | -4.45±1.04 |
| 74 | JL19 | -9.25±3.84 |
| 75 | JL20 | -3.81±2.37 |
| 76 | JL21 | 1.36±4.28 |
| 77 | JL22 | -5.11±7.35 |
| 78 | JL23 | 2.14±12.01 |
| 79 | JL24 | 0.44±1.06 |
| 80 | JL25 | -3.53±11.39 |
| 81 | JL26 | -3.23±0.22 |
| 82 | JL27 | -14.2±0.98 |
| 83 | JL28 | -1.66±0 |
| 84 | JL29 | -0.13±9.63 |
| 85 | JL30 | -3.16±1.71 |
| 86 | JL31 | -3.45±5.7 |
| 87 | JL32 | 0.25±0.48 |
| 88 | JL33 | 0.5±13.23 |
| 89 | JL34 | -1.87±1.16 |
| 90 | JL35 | -4.9±5.31 |
| 91 | JL36 | -1.6±0.66 |
| 92 | JL37 | 2.51±12.32 |
| 93 | JL38 | -1.47±1.32 |
| 94 | JL39 | 3.72±5.17 |
| 95 | JL40 | 0.23±5.61 |
| 96 | JL41 | 3.7±2.24 |
| 97 | JL42 | -4.87±4.57 |
| 98 | JL43 | -3.13±8.14 |
| 99 | JL44 | 9.43±4.41 |
| 100 | JL45 | 7.96±7.25 |
| 101 | JL46 | 3.2±2.35 |
| 102 | JL47 | 8.63±5.47 |
| 103 | JL48 | -6.04±5.95 |
| 104 | LN01 | 0.57±2.12 |
| 105 | LN02 | -1.13±3.51 |
| 106 | LN03 | -16.11±5.59 |
| 107 | LN04 | -17.93±1.73 |
| 108 | LN05 | 4.94±0 |
| 109 | LN06 | 6.58±3.12 |
| 110 | LN08 | -5.35±6.17 |
| 111 | LN09 | 6.14±4.89 |
| 112 | LN11 | -2.95±2.65 |
| 113 | LN12 | -0.29±2.57 |
| 114 | LN14 | -2.62±4.99 |
| 115 | LN15 | -0.51±5.07 |
| 116 | LN16 | 3.94±11.48 |
| 117 | LN17 | -1.4±3.03 |
| 118 | LN19 | 8.57±7.07 |
| 119 | LN20 | -0.98±2.79 |
| 120 | LN21 | 3.22±4.29 |
| 121 | LN23 | -3.15±3.91 |
| 122 | LN24 | 4.7±5.59 |
| 123 | LN31 | 0.87±6.68 |
| 124 | LN32 | -2.69±3.32 |
| 125 | LN38 | 8.17±1.56 |
| 126 | LN39 | 2.68±0.89 |
| 127 | NM01 | 2.18±0.13 |
| 128 | NM02 | -16.36±13.65 |
| 129 | NM03 | 1.07±0.66 |
| 130 | NM04 | -1.99±3.06 |
| 131 | NM05 | 2.57±5.06 |
| 132 | NM06 | -10.87±8.23 |
| 133 | NM07 | -4.47±2.28 |
| 134 | NM08 | -1.55±2.66 |
| 135 | NM09 | 16.5±14.95 |
| 136 | NM10 | 1.7±4.71 |
| 137 | NM11 | -9.47±11.61 |
| 138 | NM12 | -12.80±0 |
| 139 | NM13 | 1.98±2.92 |
| 140 | NM14 | -9.13±2.58 |
| 141 | NM15 | -11.25±2.56 |
| 142 | NM16 | 12.56±13.79 |
| 143 | NM18 | 0.67±5.32 |
| 144 | NM19 | 0.01±4.16 |
| 145 | NM20 | -0.52±1.28 |
| 146 | NM21 | 2.38±3.98 |
| 147 | NM22 | -4.96±3.43 |
| 148 | NM23 | 1.83±6.46 |
| 149 | NM24 | 8.42±8.8 |
| 150 | NM25 | -0.42±1.09 |
| 151 | NM26 | 4.94±3.36 |
| 152 | NM27 | 3.54±2.38 |
| 153 | NM28 | -2.54±4.67 |
| 154 | NM29 | -0.27±1.18 |
| 155 | SX01 | -4.06±1.41 |
| 156 | XA01 | 1.92±1.95 |
| 157 | XA02 | 4.22±2 |
| 158 | XA03 | -0.97±2.77 |
| 159 | XA04 | 1.04±0.53 |
| 160 | XA05 | 0.16±2.97 |
| 161 | XA06 | 3.34±0.63 |
| 162 | XA08 | 4.42±1.89 |
| 163 | XA10 | 0.52±6.99 |
